# ITEMAS ontology for healthcare technology innovation

**DOI:** 10.1186/s12961-019-0453-y

**Published:** 2019-05-02

**Authors:** A. Moreno-Conde, Carlos Luis Parra-Calderón, S. Sánchez-Seda, G.A. Escobar-Rodríguez, M. López-Otero, L. Cussó, R. del-Cerro-García, M. Segura-Sánchez, L. Herrero-Urigüen, N. Martí-Ras, M. Albertí-Ibarz, M. Desco

**Affiliations:** 1 Grupo de Investigación e Innovación en Informática e Ingeniería Biomédicas y Economía de la Salud, Instituto de Biomedicina de Sevilla, IBiS / Hospital Universitario Virgen del Rocío / CSIC / Universidad de Sevilla, Seville, Spain; 2 Grupo de Innovación Tecnológica, Hospital Universitario Virgen del Rocío, Seville, Spain; 3 Instituto de Investigación Sanitaria Gregorio Marañón. Centro de Investigación Biomédica en Red de Salud Mental (CIBERSAM), Madrid, Spain; 4 Institut H. del Mar D’Investigacions Mèdiques, Barcelona, Spain; 5 Departamento de Bioingeniería e Ingeniería Aeroespacial, Universidad Carlos III de Madrid, Madrid, Spain; 6 Centro Nacional de Investigaciones Cardiovasculares Carlos III (CNIC), Madrid, Spain; 7 Semicrol, Santander, Spain; 8 Ministerio de Sanidad, Servicios Sociales e Igualdad, Madrid, Spain; 9 Instituto de Investigación Sanitaria Marqués de Valdecilla, Santander, Spain; 10 Fundació Institut d’Investigació en Ciències de la Salut Germans Trias i Pujol, Barcelona, Spain; 11 IDOM Consulting, Engineering, Architecture, SAU, Bilbao, Spain; 12 Virgen del Rocío University Hospital, Avda. Manuel Siurot, s/n, 41013 Seville, Spain

**Keywords:** Ontology, innovation management, medical technology, healthcare, indicators

## Abstract

**Background:**

The Platform for Innovation in Medical and Health Technologies (ITEMAS) is a network of 66 healthcare centres focused on fostering innovation in medical and health technologies as an essential tool for increasing the sustainability of the Spanish healthcare system. The present research is focused on defining a formal representation that details the most relevant concepts associated with the creation and adoption of innovative medical technology in the Spanish healthcare system.

**Methods:**

The methodology applied is based on the methontology process, including peer-review identification and selection of concepts from the ITEMAS innovation indicators and innovation management system standards. This stage was followed by an iterative validation process. Concepts were then conceptualised, formalised and implemented in an ontology.

**Results:**

The ontology defined describes how relationships between employees, organisations, projects and ideas can be applied to generate results that are transferrable to the market, general public and scientific forums. Overall, we identified 136 concepts, 138 object properties and 30 properties in a five-level hierarchy. The ontology was tested and validated as an appropriate framework for calculating the ITEMAS innovation indicators.

**Conclusions:**

The consensus concepts were expressed in the form of an ontology to be used as a single communication format between the members of the ITEMAS network. Healthcare centres can compare their innovation results and obtain a better understanding of their innovation context based on the reasoning techniques of artificial intelligence. As a result, they can benefit from advanced analytical capabilities to define the most appropriate innovation policies for each centre based on the common experience of the large number of healthcare centres involved. The results can be used to create a map of agents and knowledge to show capabilities, projects and services provided by each of the participating centres. The ontology could also be applied as an instrument to match needs with existing projects and capabilities from the community of organisations working in healthcare technology innovation.

## Introduction

Deployment of new products and services in healthcare requires long, incremental and path-dependent innovation processes, which are strongly influenced by medical practice and developments in many different sectors, technologies and scientific disciplines [1–3]. Specialised networks composed of organisations involved in healthcare innovation have been developed worldwide to support these innovation processes. Such is the case of the organisation Consortia for Improving Medicine with Innovation and Technology, which is composed of leading institutions and hospitals such as the Massachusetts Institute of Technology, Harvard Medical Hospital, Massachusetts General Hospital, and Brigham and Women’s Hospital [4]. The consortia network has been working for 17 years as a facilitator to synergise, harmonise and synchronise the work of diverse professionals and envisions the creation of solutions for pressing unmet medical needs. In England, the National Health Service established the Academic Health Science Networks in 2013 with the aim of spreading innovation at pace and scale, improving health and generating economic growth [5]. These examples show that the traditional concept of hospitals as mere consumers of knowledge and technologies developed by universities and external companies has changed. The number of hospitals incorporating structures focused on innovation management and technology transfer is growing. This paradigm shift is based on the acknowledgement of hospitals as knowledge generators. The knowledge generated can be transferred to the market through innovations that may help to improve the sustainability of the healthcare system [6]. The implementation of innovations in the healthcare environment is influenced by multiple factors related to organisations, professionals and users, innovation facilities, procedures and socio-political context.

### Spanish context

Spain generates a large amount of scientific knowledge; however, this does not easily translate into new services and products. Similarly, it ranks ninth in science generation, although its innovation level does not match this ranking [7]. The global innovation index shows that Spain is 28th in the world ranking, and the Spanish efficiency of innovation ranks 36th [8]. New approaches are required to reduce this gap between research and innovation by overcoming the barriers associated with the development of new services and products in healthcare. Therefore, in 2010, the Carlos III Health Institute, which belongs to the Spanish Research, Technological Development, and Innovation System, founded the Platform for Innovation in Medical and Health Technologies (ITEMAS) as a novel initiative aimed at fostering innovation in Spanish healthcare centres [9]. This network is obtaining highly positive results based on the creation of Innovation Support Units in the main Spanish hospitals. Moreover, the network also addresses how to overcome the most relevant barriers associated with creating and incorporating innovation in the hospital environment. ITEMAS now comprises 67 healthcare organisations, including hospitals, healthcare centres, the Ministry of Health, and more than 100 public and private institutions focused on healthcare technology innovation (HTI).

### Measuring innovation activities in healthcare centres

A quantitative assessment of innovation activities is essential for decision-making in the field of economy and competitiveness policies. The Organization for Economic Cooperation and Development put forward a set of manuals for assessing these activities at national, regional and institutional level. The Frascati Manual presents indicators associated with research and development (R&D) activities [10]. The Oslo Manual provides guidance for collecting and using data about innovation activity in companies [11]. The Canberra Manual focuses on standardising human resources devoted to innovation [12]. Nevertheless, none of these manuals is directly applicable to healthcare centres.

To generate a set of indicators about innovation activities in Spanish healthcare centres, ITEMAS compiled a set of indicators in an innovation manual [13]. The indicators have been applied since 2014 to follow and evaluate healthcare innovation activities in ITEMAS member centres.

### Standards in innovation management systems

In 2006, the Spanish Standard Development Organization published the first Spanish standard for innovation management (*Una Norma Española* (UNE) 166000 series) [14]. This standard aims to guide organisations in the introduction, development and maintenance of frameworks for systematic innovation management practices (Innovation Management Systems). This work was a relevant input for the European Committee for Standardization, resulting in a new international technical specification for innovation management (CEN/TS 16555 Innovation Management System) [15]. This European technical specification represents a standardisation that enables companies and organisations to improve their innovation management, covering all kinds of innovation and related areas, as well as the relationship with R&D activities [15].

### Ontologies for innovation management

Information technologies have a strong impact on the organisational structure of governments, hospitals, healthcare centres and private companies. These organisations rely upon this technology for collecting, producing, representing, processing and exchanging information. They increasingly depend on information technology standards and protocols to guarantee the mechanism for information management that forms the basis of collaborative work [16]. As a result, how data are collected in information systems has a direct impact on the potential to process and exploit information within an organisation. The HTI field involves professionals from multiple knowledge areas such as medicine, engineering, economics and law. The large number of different backgrounds in this relatively new field necessitates consensus on the concepts used. During the development of the ITEMAS information system, we detected discrepancies between professionals with respect to the semantics of several terms and taxonomies. As a result, there arose a need for a mechanism to represent knowledge based on consensus between the relevant parties.

In the field of artificial intelligence, the explicit formal specifications of the terms in the domain and the relationships between them are expressed as an ontology [17]. An ontology is thus defined as a “*formal naming and definition of the types, properties, and interrelationships of the entities that really exist in a particular domain*” [18]. The definition of an ontology conveys the following benefits: (1) representing and sharing knowledge defined by consensus between multiple stakeholders, (2) defining a communication specification between multiple systems, (3) retrieving information based on semantic search engines, and (4) applying inductive reasoning mechanisms to generate inferences from the data collected. We can find examples of ontologies focused on innovation, including a small set of concepts about how innovations are proposed to solve unmet needs [19]. Other experiences focused on innovation management do not take into account existing standards for innovation management systems [20, 21]. Therefore, there remains a need to define an ontology for innovation focused on healthcare centres.

### Objective

In this study, a formal representation is defined to specify the most relevant concepts associated with HTI. The definition of concepts and semantic relationships is the basis for applying further artificial intelligence techniques that could enable us to make inferences about innovation within the framework of Spanish hospitals belonging to the ITEMAS platform.

## Methodology

The ITEMAS ontology was defined by the ITEMAS Information Management System Working Group, following the ‘methontology’ methodology, which guides the definition of ontology through a set of activities such as specifying, conceptualising, formalising, implementing and maintaining concepts [22]. The ontology focused mainly on HTI in public healthcare institutions.

### Team composition and coordination

The ITEMAS ontology was developed by the Information Management System Working Group, which comprised 13 members (3 ontology editors and 9 reviewers) and held 9 teleconferences between March and December 2017. Table 1 details the background and experience of the team members. All the 13 members participating in this research as ontology editors or reviewers have been involved in the implementation of innovation processes as part of the innovation units of the ITEMAS centres. They have also participated in the development and implementation of innovations. Platform ITEMAS has developed and implemented a Best Practices Guide in healthcare innovation management that has been published and shared among ITEMAS members, collaborators and different institutions involved in healthcare innovation (government agencies, companies, research centres, etc.).

**Table 1:** Personal details of the team members

Member characteristics	*n* (%)
Total number of members	13 (100.0%)
Female	7 (53.8%)
Profiles (more than one is allowed)	
Technical	6 (46.1%)
Life sciences	6 (46.1%)
Management	3 (23.1%)
PhD	5 (38.5%)
Experience in research, development and innovation	
Less than 5 years	1 (7.7%)
Between 5 and 10 years	3 (23.1%)
Between 10 and 15 years	2 (15.4%)
Between 15 and 20 years	5 (38.5%)
More than 20 years	2 (15.4%)

The objective of this guide is to facilitate the implementation of an innovation model that complies with the Spanish regulation and the norm for innovation management. It includes aspects such as defining a strategy, fostering a culture for innovation, innovation processes and measurement.

The analysis of the HTI field in Spanish healthcare centres was carried out based on the (1) ITEMAS Innovation Indicators, developed by ITEMAS based on previous international standards such as the Frascati [10], Oslo [11] and Canberra [12] manuals, in combination with the ITEMAS Strategic Plan, and (2) UNE 166000 standard terms and definitions for R&D and innovation management, which contains and defines 39 concepts [14].

### Task 1: Identification of concepts

During the first 2-month period, the UNE 166000 standard and ITEMAS indicators documents were divided into several sections, each of which was assigned to a working team (working teams comprised two people, with a total of five working teams participating in this task). Each month, every team member received their assigned sections and spent a period of 15 days identifying candidate concepts to be included in the ontology. Working teams determined the priority level of each concept to be included in the ontology. Ontology editors collected the identified terms in a spreadsheet and reviewed the level of consensus between multiple members of a team. In cases were no agreement was reached between the members of a team, a designated ontology editor (from the group of three) proposed the priority level in consensus with the previous working team.

### Task 2: Conceptualisation and formalisation

For 5 months, ontology editors proposed definitions for each concept and established their semantic relationships with other concepts included in the ontology. Every month the concept definitions and relationships proposed were reviewed for 15 days by team members in an iterative process until all of the working teams had reached a consensus.

#### Task 3: Implementation

The computable version of the ontology was developed in OWL with the Protégé tool and reviewed through the Protégé web browser [23].

### Task 4: Validation

The ontology developed was applied to obtain four indicators for the level of adoption of R&D and innovation in healthcare centres.

### Task 5: Maintenance

A maintenance task was established every year to ensure that the ontology was updated with emerging concepts identified with daily use of the ontology.

## Results

### Identification of concepts

**Fig. 1:**
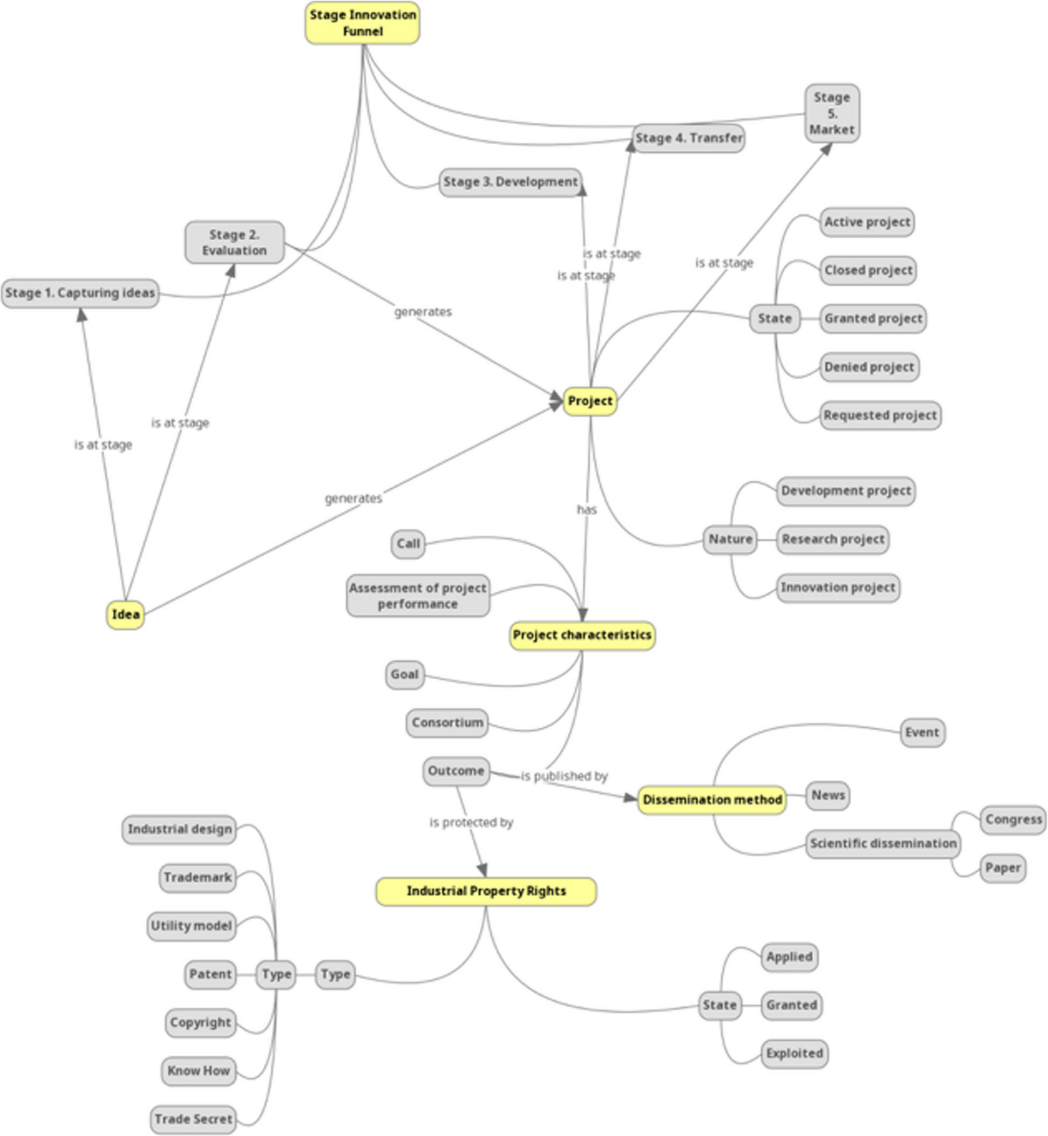
Subset of the ontology associated with the workflow from idea to transference of results

The editors divided the UNE 166000 standard [14] into sections that were assigned to the reviewers, who identified 65 candidate concepts to be included in the ontology. All these concepts were included in a spreadsheet and reviewed during a teleconference with team members and ontology editors. With consensus between editors and reviewers, all concepts were classified into three levels – high, medium and low – based on their relevance for the scope of the ontology. The 44 concepts classified as being of high or medium relevance were included in the ontology. The same process was applied to the ITEMAS innovation indicators, thus leading to the identification of 145 candidate concepts, from which 70 were considered sufficiently relevant to be included in the ontology after their revision in a teleconference with team members and ontology editors. Figure 1 represents a subset of the ontology associated with the workflow from idea to transference of results.

### Conceptualisation and formalisation

- Concept hierarchies and definitions: The ontology editors defined a hierarchy of concepts through a bottom-up strategy, starting from specific concepts that were grouped to build the general classes [24]. For example, after identifying the concepts ‘company’ and ‘institution’, it was necessary to create a generic class ‘organisation’ to cover both concepts. The application of this bottom-up strategy generated 20 additional concepts. Consequently, the final ontology comprised 134 concepts in total. Each concept received a definition from official sources (Royal Academy of the Spanish Language [25], Spanish Patent Office [26], and Spanish Government [27]).
- Object and data property relationships: A total of 136 object properties were identified to establish relationships between pairs of concepts (e.g. ‘employee’ has a relationship with ‘organisation’ through the object property ‘*worksIn*’). The concept with the most object properties was ‘project’ (26 object properties). In addition, the concept with the highest number of children was ‘IPR methods’ (18 children). Moreover, 30 data properties were defined to specify relevant attributes of identified concepts. For instance, the ‘patent’ class has the ‘PatentOffice’ property to detail where it was submitted. ‘Organisation’ was the concept with the highest number of data properties (5 data properties).
- Review process: The ontology editors prepared a document with the proposed definitions, relationships, concept hierarchies and properties to be presented to the rest of the team and assigned two reviewers for each section. This review process, which took 4 months and included a monthly teleconference for coordination, resulted in five concept definitions and one modified hierarchy.

### Implementation

The Protégé tool was applied to develop a class for each of the 134 concepts defined, and all properties, relationships and hierarchies were specified. The ontology was exported as an OWL file and uploaded to the Bioportal ontology repository [28]. Below, we detail the main sections of the defined ontology, including the description of included object and data properties in Tables 2 and 3:

1. Employee: An employee is defined as a person who works for a public or private organisation. The ontology discriminates between employees working for institutions (healthcare centres, research centres, hospitals and foundations) and those working for companies. In order to evaluate the level of innovation in public healthcare institutions, employees working in healthcare centres and research institutions include characteristics associated with their relationship with innovation such as participation as the main researcher or collaborator in R&D projects and authorship of new innovative ideas or articles published in research journals. Moreover, the position in the organisation is detailed.
2. Ideas and innovation funnel: New ideas are considered the basis for generating innovation in organisations. Ideas can evolve through the following stages: (1) idea capture, (2) valorisation, (3) development, (4) transference and (5) market. The ontology defines the relationship ‘hasIdea’ between employees and ideas, to track the generators of ideas.
3. Project: Projects are generated from ideas that receive a positive valorisation. Projects are classified based on their focus (research, development or innovation) and status (requested, granted, denied, active and closed). Projects include properties specifying the call, objectives, results and evaluation. Moreover, the set of organisations participating in the project are specified as part of a ‘consortium’ property.
4. Organisation: Organisation has properties that detail its scope (national or international) and character (public or private). The ontology defines two types of organisations, namely public healthcare institutions, which are members of the Spanish healthcare system, and private companies, which belong to the field of health R&D and innovation ecosystem in Spain. The institutions could be either healthcare centres or research institutes and generate HTI based on ideas and projects. These centres include as properties their innovation policies, goals and objectives. Technology-based enterprises, on the other hand, are a special kind of company that arise from the innovation projects carried out in public institutions.
5. Innovation Support Unit: The Innovation Support Unit is a service established in hospitals and research centres whose objective is to capture, promote and valorise the knowledge generated by the institution with the aim of using the organisation as an innovation engine that transforms knowledge into value for their own centres and for society.
6. Agreement: Agreements are applied in the innovation field between multiple parties in order to promote mutual commitment and respect for a set of conditions. The most relevant agreements in this field include exclusivity agreements, alliances, R&D contracts, transference contracts, framework agreements, donations, licenses, sponsorship, material transfer agreements and nondisclosure agreements.
7. Industrial property rights: These mechanisms provide legal protection for innovation results. The ontology includes the following intellectual property types: industrial secret, know-how, software protection, copyright, industrial design, brand, patent and utility model.
8. Reports: The ontology includes the following types of reports for projects and ideas that are in the evaluation stage: market analysis, patentability report, technical feasibility and product value report.

**Table 2:** Detailed hierarchies and number of properties for employee, project, idea, innovation funnel and organisation sections

Section	Hierarchies
Employee Object properties: 10 Data properties: 2	Employee ● Company employee ● Institution employee ○ Centre employee ■ Position ● Researcher ○ Principal investigator ○ Collaborating researcher ● Manager ○ Research institute Director ○ Hospital manager ■ Works at ISU (Innovation Support Unit) ● Director ISU
Idea and Innovation Funnel Object properties: 9 Data properties: 1	Idea Innovation Funnel ● Stage 1. Capturing ideas ● Stage 2. Evaluation ● Stage 3. Development ● Stage 4. Transfer ● Stage 5. Market
Project Object properties: 26 Data properties: 3	Project ● Stage ○ Active project ○ Closed project ○ Granted project ○ Rejected project ○ Requested project ● Nature ○ Development ○ Research ○ Innovation Project characteristics ● Call ● Assessment of project performance ● Consortium ● Goal ● Outcome
Organisation Object properties: 23 Data properties: 5	● Company ○ Technology-based company ■ Spin-off ■ Start-up ○ Pharmaceutical company ● Institution ○ Centre ■ Health centre ■ Institute for Health Research ○ Foundation

**Table 3:** Detailed hierarchies and number of properties for the Innovation Support Unit, agreement, methods of protection, methods of dissemination and report sections

Section	Hierarchies
Innovation Support Unit (ISU) Object properties: 9 Data properties: 1	ISU ISU funding ISU Units ● Research and Development (R&D) Unit ● R&D Management Unit ITEMAS
Agreements Object properties: 9 Data properties: 1	Agreement ● Exclusive agreement ● Alliance ● R&D contract ● Transfer contract ● Framework agreement ● Donation ● License ● Sponsorship ● Material transfer agreement ● Non-disclosure agreement
Industrial property rights Object properties: 7 Data properties: 1	Industrial property rights ● State ○ Applied ○ Granted ○ Exploited ● Type of protection ○ Industrial design ○ Trademark ○ Utility model ○ Patent ○ Copyright ○ Know-how ○ Industrial secret
Methods of dissemination Object properties: 3 Data properties: 1	Methods of dissemination ● Scientific dissemination ○ Congress ○ Paper ● Events ● News
Reports Object properties: 2 Data properties: 0	Reports ● Market research ● Patentability report ● Product value report ● Technical feasibility report

### Validation stage

This stage focused on identifying possible errors in the definition process and verifying the consistency of the ontology. The ontology was also tested to demonstrate its ability to be applied as a tool for calculating the ITEMAS innovation indicators.

#### Consistency

The reasoner was applied to identify inconsistencies such as rules that have contradictory or overlapping definitions. There were 13 properties that required redefinition.

#### Indicator calculation

**Fig. 2:**
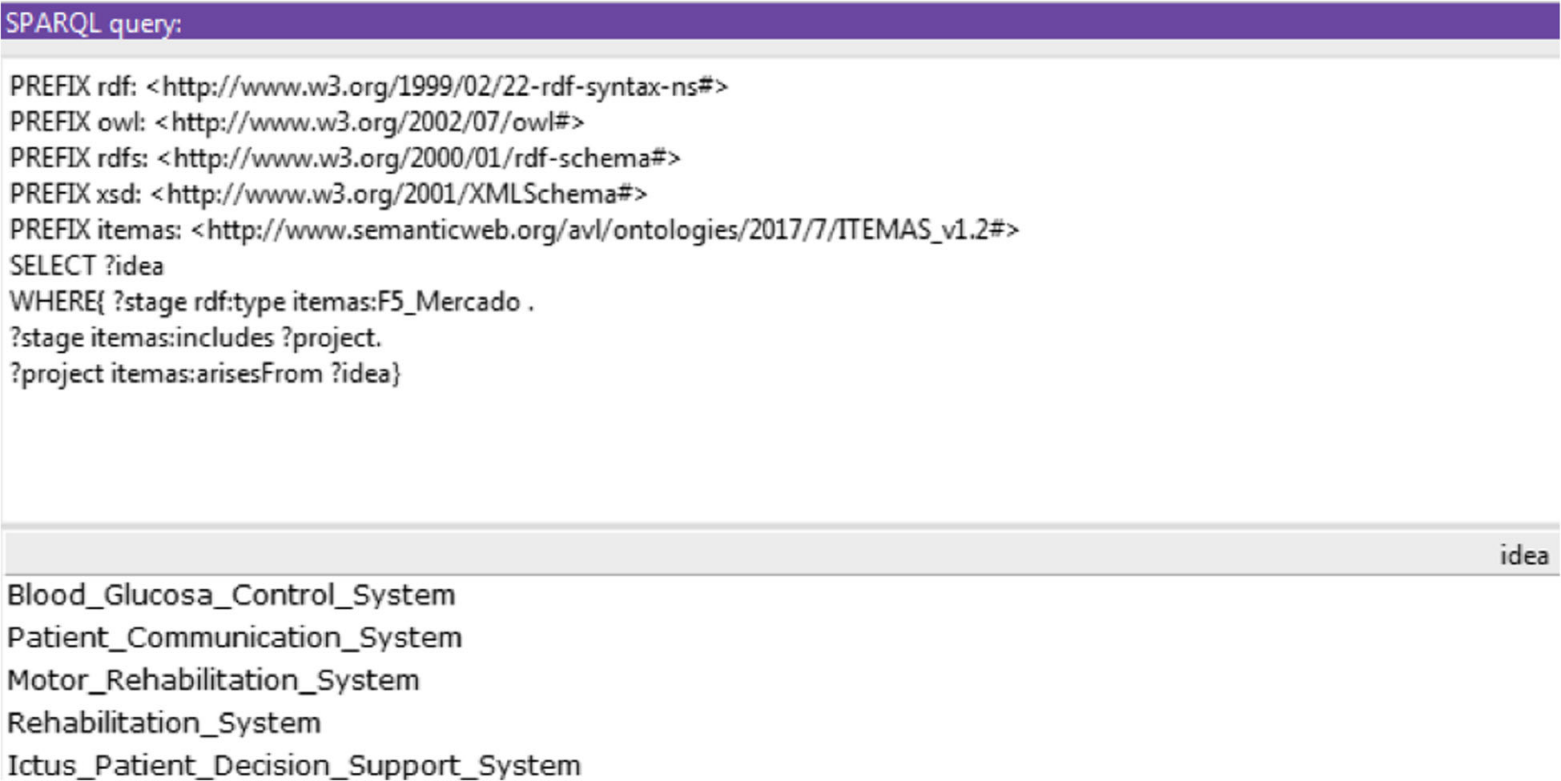
SPARQL query for calculating the list of ideas whose results were transferred to the market

The SPARQL language (Protocol and RDF Query Language) was applied to calculate the ITEMAS innovation indicators associated with environment, processes, resources and results. To avoid sharing private data in this publication, the ontology was populated with dummy data about the level of innovation in multiple health centres. As an example, Fig. 2 details the SPARQL queries defined to calculate the list of ideas that generated projects with results that had already been transferred to the market (stage 5). The query returns the list of five projects that generated products already transferred to the market.

### Maintenance

**Fig. 3:**
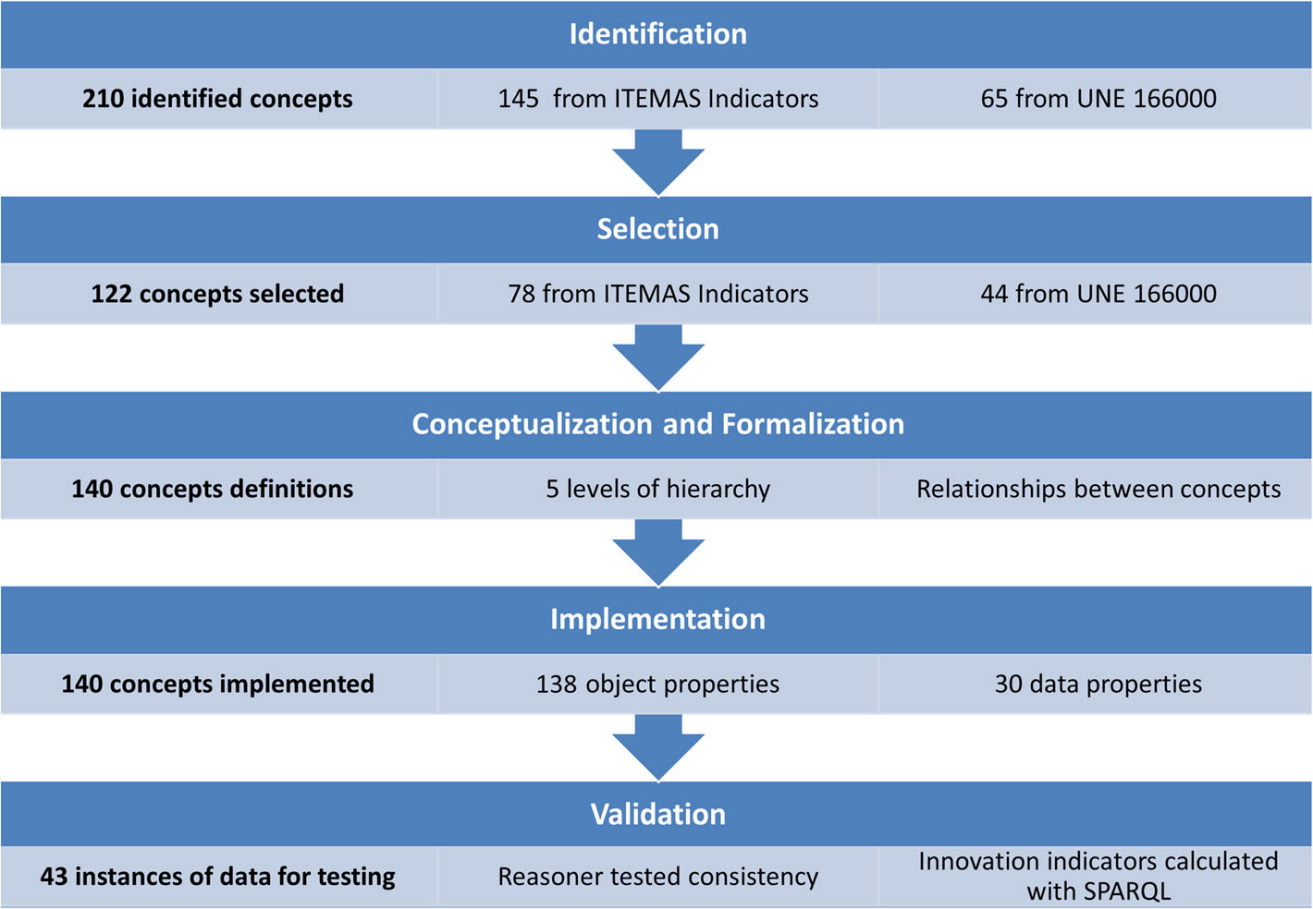
Summary of the methodology applied

The ontology is updated regularly during its established lifetime based on the inputs collected by the Information Management System Working Group. The ontology is expected to evolve based on newer innovation indicators that might be defined in the future. Figure 3 summarises the ontology modelling process and details how the number of concepts evolved through the steps of the ‘methontology’ scheme.

## Discussion

To our knowledge, the ITEMAS ontology is the first attempt to focus on representing concepts and semantics associated with HTI in the Spanish healthcare system. The ontology defined is based on the needs identified from 66 centres that are already collecting this information to measure their level of innovation. The scope of the ontology enables us to identify how the multiple ideas generated in healthcare centres evolve towards generating new products and services. Moreover, the ontology includes concepts associated with management of innovation, resources, employees, dissemination, and intellectual and industrial property.

The ontology is aligned with the best practices in innovation management, since the concepts addressed have been applied for many years. They have therefore reached an appropriate level of maturity and have been homogenised according to applicable standards for innovation management. Since most concepts of ITEMAS ontology were extracted from the UNE 166000 standard, it is expected that core concepts of the ontology will remain persistent for several years (standards have a review cycle every 5 years).

The ontology modelling process focused on avoiding the dependence on personal opinions through peer review of each step of the methontology. Concepts were defined based on the Royal Academy of the Spanish Language and information from governmental institutions to ensure the inclusion of official definitions. As a result, our ontology is expected to contribute to a consensus in the definition of concepts in the HTI field. This is especially relevant in the innovation field, where there is a lack of consensus about the boundaries between concepts associated with either innovation or research. Moreover, HTI is clearly based on collaboration between multidisciplinary teams composed of professionals with medical, technological, legal and other technical background that requires homogeneous definitions. Our approach is based on existing standards in order to reach consistent definitions for those concepts applicable to the multiple fields involved in HTI.

Adoption of standardised innovation management in healthcare centres results in consistent data collection that may contribute to better decision-making within the Spanish healthcare system. Moreover, the ontology defined allows us to apply semantic web techniques for analysis of collected data. Inferences based on ITEMAS innovation indicators could lead to monitoring the impact of R&D policies in the participating institutions. As an example, healthcare institutions will be able to determine the impact of creating an Innovation Support Unit on the number of projects, publications, and transferred products and services.

In the coming year, ITEMAS intends to create a map of agents and knowledge to show the capabilities and projects of the Spanish healthcare system, as well as the services provided by its participating centres. The ontology could be applied to match needs with existing projects and capabilities from the community of organisations interested in HTI. The ontology will be the cornerstone for future services that will establish relationships between the multiple actors involved in the innovation process and the results. Data collected through the ITEMAS portal and provided by members will be applied to identify and highlight best practice in the HTI field. As a result, this ontology is expected to contribute to the promotion of new collaborations and innovation initiatives between members of the ITEMAS network. Moreover, this ontology can be applied as a technical specification that will enable information to be shared between IT systems from multiple healthcare centres, thus contributing to the creation of an open market for developing software focused on healthcare innovation management. Although the ITEMAS ontology is led by Spanish healthcare centres, it also addresses a need existing at an international level since HTI is an area of global relevance. On the one hand, ITEMAS is growing beyond the Spanish borders, with healthcare centres from Spanish-speaking countries such as Colombia and Venezuela having already joined the network. On the other hand, it is expected that translations of the ontology to other languages could be applicable to healthcare centres at an international level.

Moreover, it is precisely the high-level conceptualisation based on ontologies that allow the extension/modification of the conceptual model to adapt it to other contexts.

Healthcare centres and actors involved in HTI will be able to obtain a consistent management of information based on the defined concepts and its relationships. Moreover, translated versions of the ITEMAS ontology concepts could provide an opportunity for cross-border collaboration with companies and hospitals from different countries. This is especially relevant nowadays with the globalisation of the healthcare market. In this regard, the European Commission is working towards the establishment of a Digital Single Market across multiple European Member States.

### Limitations

The ontology is asymmetrical, as it represents concepts from the perspective of healthcare centres and does not include the same level of detail for personnel structures and levels of adoption of innovation in companies collaborating with these centres. ITEMAS plans to define new innovation indicators specifically designed to represent the innovation characteristics of the collaborating companies. These indicators might be incorporated in future versions of the ontology to obtain a more complete representation of companies collaborating in this field. In addition, the authors recognise that, since the ontology concepts were included primarily in the context of innovation in Spanish healthcare centres, translations to other languages or countries would require the exact definition of each specific term to be verified. The lack of standards may cause some misunderstanding between languages. For instance, ‘research institute’ in Spain is the term used to refer to those research centres that arise from collaboration between hospitals and universities to promote translational research. When the term is translated directly to English, it loses its meaning. In this respect, the International Organization for Standardization is currently working on the draft ISO 50500 standard on innovation management terminology, which will provide globally consistent definitions of innovation terms [29]. Harmonisation with this standard will be required for wide and unambiguous adoption at international level of the concepts included in the ITEMAS ontology.

## Conclusions

Our research constitutes the first approach to focus on formally representing the concepts associated with HTI in the Spanish healthcare system through its centres. This research has led to consensus on the definition of concepts associated with measuring the level of innovation based on organisational environment, processes, resources and results. The concepts agreed upon were expressed in the form of an ontology so that they would facilitate specific communication between the 66 centres included in the ITEMAS network. The defined interoperable specification will enable data to be combined from multiple sources in real time from those centres interested in sharing their innovation metrics. Healthcare centres will therefore be able to compare their innovation results and obtain a better understanding of their innovation context based on artificial intelligence reasoning techniques. This initiative is open for additional international collaborations, thus providing an opportunity to establish an international network of companies and hospitals from various countries.

## Abbreviations

HTI: Healthcare Technology Innovation
ITEMAS: Platform for Innovation in Medical and Health Technologies
R&D: Research & Development
UNE: *Una Norma Española*
